# Collaborative Excellence for Public Health Dementia Education

**DOI:** 10.1093/geroni/igaf122.2218

**Published:** 2025-12-31

**Authors:** John Shean, Elma Johnson, Mickal Lewis, Alexandra Nordyke, Madiha Shakir, Kayleigh Jones, Eva Jackson

**Affiliations:** Alzheimer’s Association, Chicago, Illinois, United States; University of Minnesota, Minneapolis, Minnesota, United States; Alzheimers Association, Chicago, Illinois, United States; New York University Langone Health, New York, New York, United States; Ullu Learning Design, LLC, Atlanta, Georgia, United States; Emory University, Atlanta, Georgia, United States; Alzheimer’s Association, Chicago, Illinois, United States

## Abstract

As the prevalence of Alzheimer’s disease and related dementias continues to increase, public health action is necessary across the primary, secondary and tertiary levels of prevention. In 2020, the Centers for Disease Control and Prevention (CDC) funded three Building Our Largest Dementia Infrastructure (BOLD) Public Health Centers of Excellence to focus on these three levels. Working with the three Centers of Excellence, the Alzheimer’s Association developed and released modules as part of a free, online, interactive public health curriculum for current and future public health professionals. In this session, attendees will learn more about A Public Health Approach to Dementia, including the 3-part curriculum suite focused on the prevention levels and how this resource can be used by faculty and students. This suite explores and addresses the following topics: Public Health and Dementia Risk Reduction; Public Health and Early Detection of Dementia and Diagnosis; and Public Health and Dementia Caregiving. Attendees will learn about the innovative applications of a virtual, self-paced curriculum that incorporates learner-centered user experience design principles. The curriculum can be used as stand-alone modules or as a suite. Attendees will also experience use of the instructor guide that enhances virtual and classroom learning experiences. Dementia is a public health issue because the prevalence and costs are substantial, the impacts are serious and there are public health solutions. Now is the time to use innovative, collaborative methods such as this curriculum suite to equip the current and future public health workforce.

